# Cx43 Mediates Resistance against MPP^+^-Induced Apoptosis in SH-SY5Y Neuroblastoma Cells via Modulating the Mitochondrial Apoptosis Pathway

**DOI:** 10.3390/ijms17111819

**Published:** 2016-11-01

**Authors:** In-Su Kim, Palanivel Ganesan, Dong-Kug Choi

**Affiliations:** 1 Department of Biotechnology, Konkuk University, Chungju 380-701, Korea; kis5497@kku.ac.kr; 2 Nanotechnology Research Center and Department of Applied Life Science, College of Biomedical and Health Science, Konkuk University, Chungju 380-701, Korea; palanivel@kku.ac.kr

**Keywords:** connexin 43, mitochondira, apoptosis, SH-SY5Y, 1-methyl-4-phenylpyridine (MPP^+^)

## Abstract

Neuronal apoptosis in the substantia nigra par compacta (SNpc) appears to play an essential role in the pathogenesis of Parkinson’s disease. However, the mechanisms responsible for the death of dopaminergic neurons are not fully understood yet. To explore the apoptotic mechanisms, we used a well-known parkinsonian toxin, 1-methyl-4-phenylpyridine (MPP^+^), to induce neuronal apoptosis in the human dopaminergic SH-SY5Y cell line. The most common method of interaction between cells is gap junctional intercellular communication (GJIC) mediated by gap junctions (GJs) formed by transmembrane proteins called connexins (Cx). Modulation of GJIC affects cell viability or growth, implying that GJIC may have an important role in maintaining homeostasis in various organs. Here, we hypothesized that increasing the level of the gap junction protein Cx43 in SH-SY5Y neuroblastoma cells could provide neuroprotection. First, our experiments demonstrated that knocking down Cx43 protein by using Cx43-specific shRNA in SH-SY5Y neuroblastoma cells potentiated MPP^+^-induced neuronal apoptosis evident from decreased cell viability. In another experiment, we demonstrated that over-expression of Cx43 in the SH-SY5Y cell system decreased MPP^+^-induced apoptosis based on the MTT assay and reduced the Bax/Bcl-2 ratio and the release of cytochrome C based on Western blot analysis. Taken together, our results suggest that Cx43 could mediate resistance against MPP^+^-induced apoptosis in SH-SY5Y neuroblastoma cells via modulating the mitochondrial apoptosis pathway.

## 1. Introduction

Apoptosis, a type of cell death, plays an important role in early development and in the growth of normal adult tissues [1,2]. It is also important for organogenesis and synapse formation in the central nervous system (CNS) during development. During normal development of the CNS, it is a common phenomenon [3,4]. However, the survival of the neurons is more necessary in the adult brain for their normal function across their entire lifetime. This helps in the normal functions of the neuronal cells within their circuits. During many disease conditions or injuries, excessive loss of neurons or neuronal death is seen in many patients. The neuronal loss or death is seen most commonly in CNS-related diseases such as stroke, Alzheimer’s, Parkinson’s and Huntington diseases.

Parkinson’s disease (PD) is one of the most common neurodegenerative disorders. The prevalence of PD ranks it second, only lower than Alzheimer’s disease. PD is characterized by the tetrad of akinesia, rigidity, tremor at rest, and postural instability. PD is a slowly progressing neurodegenerative disorder without a clear etiology. Pathological hallmarks of PD include the death of dopaminergic neurons of the substantia nigra (SN) and the presence of Lewy bodies (α-synuclein with ubiquitin positive, eosinophilic, and cytoplasmic inclusions) in the surviving neurons. A recent etiological study in twins [5] strongly suggests that environmental factors play an important role in typical non-familial PD patients over the age of 50 years. It has been reported that environmental factors can increase the risk of PD development, including rural living, well water use, and exposure to chemicals (pesticides, herbicides, industrial chemicals, and 1-methyl-4-phenyl-1,2,3,6- tetrahydropyridine (MPTP)) [6]. To study neural degeneration in PD, neurotoxin 1-methyl-4-phenylpyridinium (MPP^+^), the active metabolite of MPTP, has been widely used because it causes a severe Parkinsonian-like syndrome with loss of dopaminergic cells through selectively and potently inhibiting complex I of the mitochondrial electron transport chain in both cellular and animal models [7,8,9].

Gap junctional intercellular communication (GJIC) modulation changes can affect the cell growth and viability of the neural cells. Homeostasis of various organs including the brain is influenced by the GJIC, which can effectively control the neural cellular pH, glutamate and K^+^ in the brain cells [10,11,12]. Connexin43 (Cx43) is an important member of the family of gap-junction proteins that is widely expressed in various tissues and organs of the vertebrates, including humans [13]. Cx43 also regulates various cellular functions, including cell growth and differentiation, cell migration, and cell survival [14]. Altered Cx43 expression has been recently observed in many CNS-related diseases which can either increase or decrease Cx43 or GJIC of the injured brain cells [15,16,17].

In this study, we determined whether there were variations in the expression level of Cx43 during MPP^+^-induced apoptosis and whether a similar response was elicited in H_2_O_2_- and 6-OHDA–treated SH-SY5Y cells. Mitochondria-mediated cell death was studied along with alterations in the Cx43 level during MPP^+^-induced apoptosis. In addition, the effects of silencing and overexpression of the Cx43 level on MPP^+^-induced apoptosis were determined.

## 2. Results

### 2.1. Expression of Connexin 43 (Cx43) Is Suppressed in Apoptotic SH-SY5Y Cells

**Figure 1:**
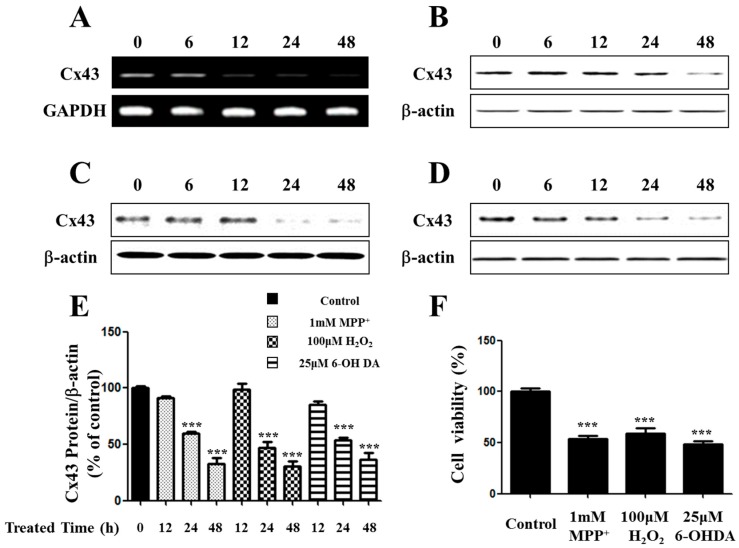
Expression of Cx43 on toxin-treated SH-SY5Y cells. SH-SY5Y cells were treated with MPP^+^ for 6 to 48 h. The levels of Cx43 were measured by RT-PCR (**A**) and immunoblot (**B**); Cells were treated with 100 µM H_2_O_2_ (**C**) and 25 µM 6-OHDA (**D**) for 0 to 48 h. Cell lysate samples (20 µg protein/lane) were measured by immunoblot analysis using Cx43 antibody; (**E**) Bar graphs showing quantitative data of Cx43 protein levels were normalized to the level of β-actin; (**F**) Cell viability of SH-SY5Y treated with toxins for 48 h. *** *p* < 0.001 compared with control group in one-way ANOVA followed by Bonferroni post hoc test.

Connexin expression can enhance apoptosis induction in a connexin-dependent manner [18]. Among different isoforms of connexin molecules, connexin 43 (Cx43) has been extensively investigated because of its ubiquitous expression in a variety of cell types. To determine the expression of Cx43 in MPP^+^-induced apoptotic SH-SY5Y cells, we analyzed expression of Cx43 for treatment times. Results indicated that the expression of Cx43 was significantly suppressed in MPP^+^-induced apoptosis. The expression levels of Cx43 mRNA and protein were down-regulated in a time-dependent manner (Figure 1A,B).

Other toxins such as hydrogen peroxide (H_2_O_2_) and 6-OHDA can also cause oxidative stress and apoptosis. H_2_O_2_ and 6-OHDA have strong oxidizing properties. They can result in the generation of reactive oxygen species (ROS). Oxidative stress resulting from an imbalance of aerobic metabolism can impose a serious threat to cellular homeostasis. High levels of ROS will oxidize lipids, proteins, and DNA, leading to tissue damage and cell death [19]. Several recent studies have reported that ROS is deeply involved in the pathophysiology of several neurodegenerative diseases, including Alzheimer’s disease and Parkinson’s disease (PD) [20,21]. In addition, ROS can alter mitochondrial function and mitochondrial permeability transition pores [22,23].

Following treatment with 100 µM H_2_O_2_ and 25 µM 6-OHDA for up to 48 h, neuronal cell loss was observed in almost half of the cells based on the MTT assay, similar to MPP^+^-induced cell death (Figure 1E). Cx43 protein was down-regulated in a time-dependent manner on toxin-treated SH-SY5Y cells (Figure 1C,D), similar to that in MPP^+^-treated cells (Figure 1E).

### 2.2. Knockdown of Cx43 Increases Sensitivity to MPP^+^ While Overexpression of Cx43 Reduces MPP^+^-Induced Apoptosis in SH-SY5Y Cells

**Figure 2:**
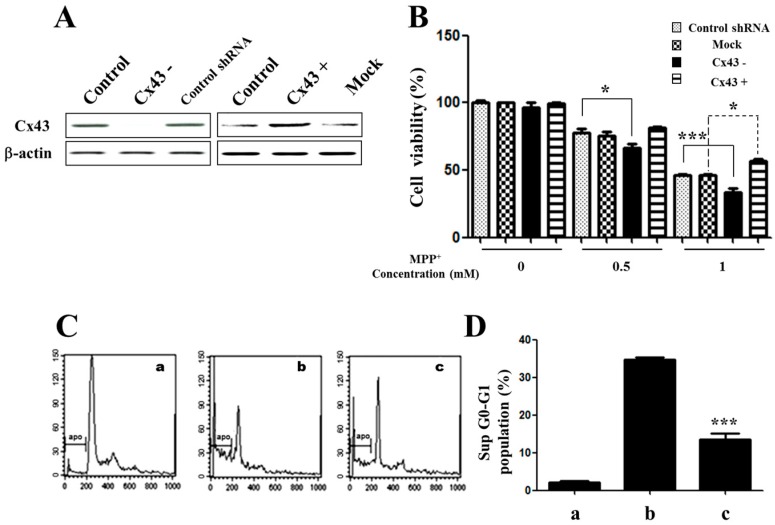
Cx43 influences the apoptosis of human SH-SY5Y cells. (**A**) Western blot showing the level of Cx43 protein in SH-SY5Y expressing shRNA-control, shRNA-Cx43, control-vector, or overexpression-Cx43; (**B**) Cell viability of SH-SY5Y expressing control, shRNA-Cx43, or overexpression of Cx43 treated with 0.5 and 1 mM MPP^+^ for 48 h; (**C**) Cytograms showing the proportion of apoptotic cells after treatment with 1 mM MPP^+^ for 60 h based on FACs analysis with PI staining; Bar graphs (**D**) showing the quantitative data of sub-G0–G1 apoptotic populations: (a) Control cells; (b) cells exposed to 1 mM MPP^+^ alone; (c) the overexpression-Cx43 cells exposed to 1 mM MPP^+^. Bar (├┤) represents sub-G0/G1 or hypodiploid DNA fraction. * *p* < 0.05, *** *p* < 0.001 compared with MPP^+^-treated group in one-way ANOVA followed by Bonferroni post hoc test.

To determine whether MPP^+^ resistance could be mediated by Cx43, endogenous Cx43 in SH-SY5Y was knocked down with Cx43-shRNA (Figure 2A). A control shRNA vector was also included in the assay. Reduced expression of Cx43 in these cells resulted in a significant increase of sensitivity to MPP^+^ treatment at concentrations ranging from 0.5 mM to 1 mM (Figure 2B), confirming a significant role of Cx43 in MPP^+^ resistance in SH-SY5Y cells.

We characterized the viability and apoptosis of MPP^+^-induced SH-SY5Y cells. Overexpression of Cx43 in SH-SY5Y cells resulted in a significant increase in the percentage of cell viability in the presence of 0.5 mM to 1 mM of MPP^+^ (Figure 2B,C). The protective role of Cx43 in MPP^+^-induced apoptosis in SH-SY5Y cells was confirmed by PI staining. DNA content histograms of cells after exposure to 1 mM MPP^+^ are shown in Figure 2C. When cells were incubated in medium alone, a typical single peak of nuclei with diploid DNA content was observed (Figure 2C) and there was only about 2%~3%. In the presence of 1 mM MPP^+^, a characteristic hypodiploid DNA content peak indicative of sub-G0–G1 apoptotic populations was detected. With overexpression of Cx43, the proportion of apoptotic cells was reduced (Figure 2C,D).

### 2.3. Overexpression of Cx43 Inhibits MPP^+^-Induced Cell Death by Increasing Mitochondrial Membrane Potential (∆Ψm) Mitochondria are Primary Target in Apoptosis

**Figure 3:**
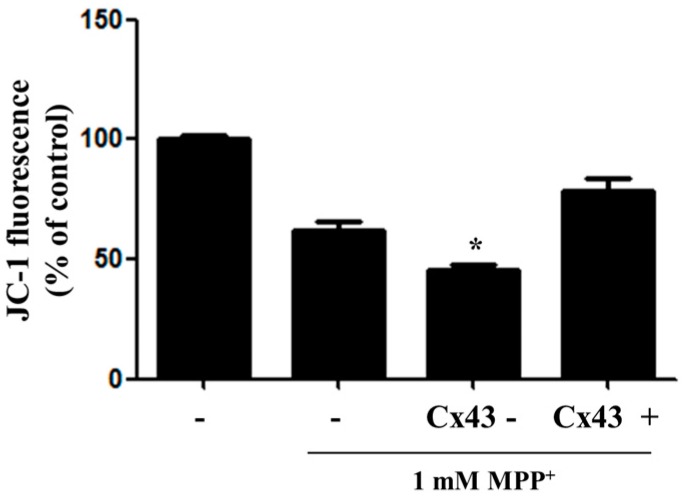
Overexpression of Cx43 inhibits MMP loss in MPP^+^-induced apoptosis. Cells were treated with 1 mM MPP^+^. MMP reduction was analyzed by JC-1 dye assay after 48 h of treatment with 1 mM MPP^+^. Cx43-: shRNA-Cx43, Cx43+: overexpression-Cx43; * *p* < 0.05 compared with MPP^+^-treated group in one-way ANOVA followed by Bonferroni post hoc test.

The disruption of mitochondrial transmembrane potential (∆Ψm) is one of the early changes in mitochondria-mediated apoptosis. Earlier reports have indicated that the disruption of mitochondrial membrane potential is involved in MPP^+^-induced neuronal cell death. To determine the effect of MPP^+^-induced neuronal cell death on the mitochondrial function of Cx43, each group was stained with JC-1 to detect ΔΨm, an indicator of mitochondrial activity. The effect of MPP+ on mitochondrial function is shown in Figure 3. Treatment with 1 mM MPP^+^ significantly reduced the mitochondrial membrane potential of normal control cells. When cells with Cx43 depletion (knockdown) were treated MPP^+^, ΔΨm was significantly decreased. However, when cells with Cx43 overexpression were treated MPP^+^, ΔΨm was significantly increased (Figure 3).

### 2.4. Bax/Bcl-2 Ratio Is Increased in SH-SY5YCells with Reduced Cx43 Level after MPP^+^ Treatment

**Figure 4:**
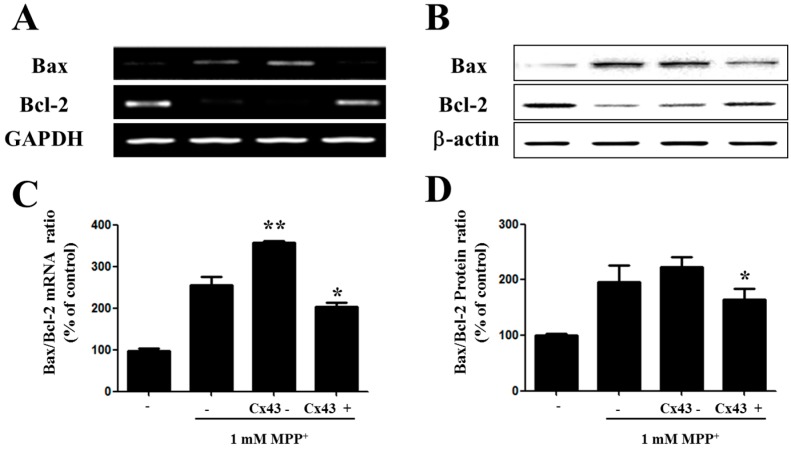
Cx43 influences the expression of the Bcl-2 family in SH-SY5Y cells. (**A**) RT-PCR showing Bax, Bcl-2 and Glyceraldehyde 3-phosphate dehydrogenase (GAPDH) expression in Cx43-knockdown (shRNA) and Cx43-expressing (Cx43) SH-SY5Y cells after 1 mM MPP^+^ treatment for 24 h; (**B**) Bax/Bcl-2 mRNA ratio after treatment of MPP^+^ for 24 h; (**C**) Western blot analysis of Bcl-2 family in Cx43-knockdown (shRNA) and Cx43-expressing (Cx43) SH-SY5Y cells after treatment with 1 mM MPP^+^ for 48 h; (**D**) Bax/Bcl-2 protein ratio after treatment of MPP^+^ for 48 h. GAPDH was used as the internal controls for RT-PCR analysis. * *p* < 0.05, ** *p* < 0.005 compared with MPP^+^-treated group in one-way ANOVA followed by Bonferroni post hoc test.

Following, we determine the role of Cx43 in the protection of cells from MPP^+^-induced apoptosis in the Bcl-2 family. The exact mechanism in which the Bcl-2 family is involved in the mitochondrial apoptotic pathway has not been fully clearly defined elsewhere. However, in certain studies the Bcl-2 family showed an important role in the mitochondrial apoptotic pathway [24]. The two important members of the Bcl-2 family are Bax and Bcl-2, which play an important role in the permeability of the mitochondrial membrane. Pore-forming cytoplasmic protein Bax enhances the release of cytochrome C from the space of the intermembrane to the cytosol which leads to the apoptosis in the mitochondrial cells [25]. In an opposite way, Bcl-2 inhibits apoptosis by preventing the release of cytochrome C by stabilizing the membranes’ permeability; thereby the mitochondrial cellular integrity was maintained [26]. In our study, RT-PCR analysis and immunoblot analysis revealed Bcl-2 at a higher level and, alternatively, Bax at a lower level in Cx43-expressing cells of MPP^+^-treated cells (Figure 4A,B). Following that, we also checked the ratio of Bax/Bcl-2 to study the susceptibility to apoptosis in these cells (Figure 4C,D). The Bax/Bcl-2 ratio was significantly higher in cells lacking Cx43 treated with 1 mM MPP^+^. These results suggest that Cx43 plays a role in preventing apoptosis via the mitochondrial-dependent pathway.

### 2.5. Cx43 Prevents Cytochrome C Release, Caspase-3 Activity, and PARP Proteolysis by Mitochondria

**Figure 5:**
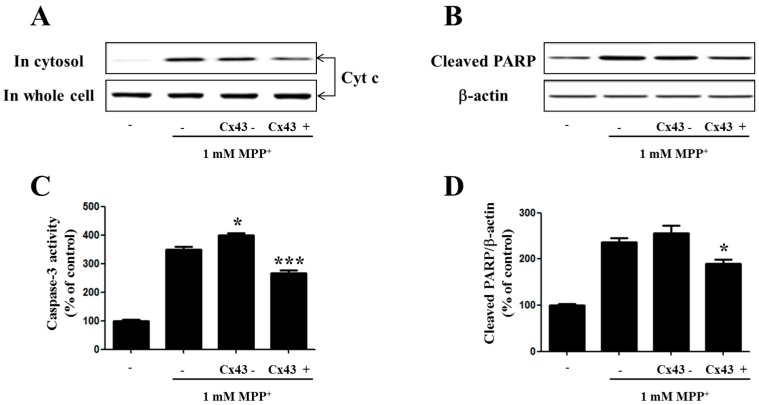
Cx43 influences the mitochondria control of the cell death pathway in SH-SY5Y cells. (**A**) Western blot analysis of cytosolic cytochrome c in Cx43-knockdown (shRNA) and Cx43-expressing (Cx43) SH-SY5Y cells after treatment with 1 mM MPP^+^ for 24 h; (**B**) Caspase-3 activity was measured by colorimetric assay kit (Sigma, CASP3P); PARP proteolysis (**C**) was measured by immunoblot analysis; Bar graphs (**D**) showing quantitative data of PARP levels normalized to the level of β-actin. * *p* < 0.05, *** *p* < 0.001 compared with MPP^+^-treated group in one-way ANOVA followed by Bonferroni post hoc test.

Mitochondria play an important role in the regulation of cell apoptosis. Cytochrome C released from mitochondria initiates the change in the membrane potential loss of the mitochondria which, in parallel, activate the caspases within the cytoplasm [27]. It has been demonstrated that caspases are important factors that trigger apoptosis. Caspase-3 is a crucial biomarker of neuronal apoptosis. It also acts as an apoptotic executor [28,29]. To better understand the influence of Cx43 on MPP^+^-induced apoptosis and cytochrome C release, as well as caspase-3 activity, cells with Cx43 knockdown by shRNA and Cx43 overexpression were exposed to 1 mM MPP^+^ for 48 h. Experimental treatment of SH-SY5Y cells with MPP^+^ demonstrated that cells with Cx43 knockdown presented a significant increase of cytochrome C loss and caspase-3 activity in the mitochondria (Figure 5), indicating that Cx43 expression in SH-SY5Y cells could inhibit MPP^+^-induced cell death by modulating the mitochondrial apoptotic pathway.

## 3. Discussion

Mitochondria serve as an intracellular powerhouse and their main role involves the energy production through the mitochondrial respiratory chain and maintaining cell apoptosis [30]. The mitochondrial functional alterations play a critical role in PD pathogenesis. The main functional alterations such as mitochondrial complex I of the respiratory system’s dysfunctions may result in reducing ATP synthesis, and neuronal degeneration in PD. As a dopamine deficiency syndrome, PD is one among the late-onset neurodegenerative disorders which results in the sustained and constant loss of the dopaminergic neurons among elderly people. Human dopaminergic SH-SY5Y cells contain good qualities of human neurons. Therefore, they have been well used to study PD [31,32,33]. MPP^+^-induced cell death is a useful representation to study dopaminergic degeneration of PD both in vitro and in vivo [34,35,36,37].

Communication between the cells is most commonly done through gap junctional intercellular communication (GJIC) with gap junctions (GJs) from transmembrane proteins called connexins (Cx). The two cells’ cytoplasms are connected through the hexameric unit of connexins in one cell (a connexin) by a couple with a corresponding connexin in a contiguous cell [38]. The alterations in the GJIC affect the cell growth and viability, which indicates GJIC might play a critical role in homeostasis regulation in various organs [10]. Many studies have reported that GJIC is very crucial for the homeostatic regulation of extracellular K^+^, glutamate and pH levels in the brains cells [11,12]. These junctions play an important role in the communication of various signals such as electrical and chemical signals for maintaining the neuro-cellular activities and survival of the cells in the neurovascular unit [39,40]. The alteration or mutation in the Cx protein may lead to various congenital diseases and most common among them is hearing loss linked to the Cx protein family [41]. In disease conditions, these gap junctions can transmit the injury to neighboring healthy cells which can enhance the injury surface leading to bystander effects [42,43]. In consequence, these junctions can serve protection by supplying essential nutrients and other metabolites and limiting the spreading of the injury to other healthy cells [13].

Our results showed a correlation between Cx43 expression and MPP^+^-induced apoptosis. Our results also indicated that Cx43 could affect the mitochondrial apoptotic pathway in the presence of MPP^+^. Some researchers have suggested that Cx43-mediated GJIC accounts for its apoptosis effects. However, GJIC has also been associated with anti-apoptotic effects by surpassing the necessary metabolites and other drug molecules for growth and serving in the bystander effect. [44,45,46,47]. Recent identification of Cx43 in the mitochondria suggests that Cx43 may participate in mitochondrial signaling, including apoptosis [48,49]. Investigation of mPTP function in SH-SY5Y cells with simultaneous measurement of the transmembrane potential has revealed that Cx43 depletion can induce mPTP opening. The opening of the mPTP in turn leads to the release of proapoptotic factors and initiates programmed cell death. The increases in the relative ratio of Bax/Bcl-2 expression, cytochrome C release, and caspase-3 activity, with decreasing levels of Cx43 in SHSY5Y cells exposed to MPP^+^ confirming that Cx43 is linked to mitochondrial signaling and apoptotic response.

In summary, this study confirmed that mitochondria-mediated apoptotic events in Cx43-depleted neuroblastoma cells include MMP loss, the release of cytochrome c into the cytosol, and the activation of caspase-3. The translocation of Bax into the mitochondria suggests Bax-mediated pro-apoptotic mitochondrial changes in the brain cells. Taken together, our results suggest that Cx43 could mediate resistance against MPP^+^-induced apoptosis in SH-SY5Y neuroblastoma cells via modulating the mitochondrial apoptosis pathway.

## 4. Materials and Methods

### 4.1. Materials

MPP^+^ and 5-diphenyl-tetrazolium bromide (MTT) were obtained from Sigma Aldrich (St. Louis, MO, USA). Culture plates (six and 96 well) and culture dishes (100 mm) were purchased from Nunc Inc. (North Aurora Road, Naperville, IL, USA). Fetal bovine serum (FBS) was purchased from Gibco-BRL Technologies (Rockville, MD, USA). Propidium iodide (PI) was supplied by BD Clontech (Terra Bella Ave, CA, USA). Antibodies against Connexin 43 (#3512), Bax (#2772), Bcl-2 (#2872), cleaved PARP (#5625) and β-actin (#3700) were obtained from Cell signaling Co. (Boston, MA, USA).

### 4.2. Cx43 Expression Plasmid Construction

To construct the pcDNA 3.1-Cx43 recombinant vector, the open reading frame (ORF) of human Cx43 cDNA was amplified using specific forward 5’-TGGCTAGCACATGGGTGACTGGA-3’ and reverse 5’-ACCTGGAGATCTAGATGGATCC-3’ primers. Two restriction sites NheI and BamHI were added at both end of the gene respectively. The Cx43 gene was then cloned into the restriction sites of EcoRI and BamHI of the expression vector pcDNA3.1.

### 4.3. Cell Culture and Transfection

The human dopaminergic SH-SY5Y cells cultured in DMEM were supplemented with 10% (*v*/*v*) inactivated FBS and 100 U/mL penicillin/streptomycin. These cells were maintained in 37 °C incubator supplied with 5% CO_2_ and 95% humidified air. Connexin 43 shRNA plasmid and control shRNA plasmid were obtained from Santa Cruz Biotechnology. They were diluted in nuclease free water to a final concentration of 10 µM. Plasmids (pcDNA 3.1-Cx43 recombinant vector, Cx43 shRNA plasmid) were transfected into SH-SY5Y cells at a final total concentration of 15 nM using Transfection Reagent (Lipofectamine 2000 (Invirogen, Carlsbad, CA, USA)). Subsequent measurements were performed at 48 h post transfection.

### 4.4. Assessment of Cell Viability

The viability of the cells was measured using MTT assay as previously described [50]. Briefly, SH-SY5Y cells seeded at 5 × 10^4^ cells/well in 96-well plate, were treated with toxins for 48 h. MTT (0.5 mg/mL) dissolved in phosphate-buffered saline was added at the end of incubation. The crystals formed by viable cells were measured were measured at wavelength of 550 nm using a microplate reader (Molecular device, Sunnyvale, CA, USA).

### 4.5. Isolation of Total RNA and Expression Analysis

Total RNA was extracted from SH-SY5Y cells (1 × 10^6^ cells/well) using TRIzol reagent (Invitrogen, Carlsbad, CA, USA). For RT-PCR, 2.5 µg of total RNA was reverse transcribed using a First-Strand cDNA Synthesis kit (Invitrogen). PCR was then performed using the above-prepared cDNA as a template. The following primers were used for PCR, Cx43 sense, 5′-CGCTCCCCTCTCGCCTATGT-3′; Cx43 anti-sense, 5′-CTGGCACGACTGCTGGCTCT-3′; Bcl-2 anti-sense, 5′-CCCAGCCTCCGTTATCCTGG-3′; Bcl-2 sense, 5′-ACGACTTCTCCCGCCGCTAC-3′; Bax sense, 5′-CACCAAGGTGCCGGAACTGA-3′; Bax anti-sense, 5′-AATGCCCATGTCCCCCAATC-3′; GAPDH sense, 5′-GCAGTGGCAAAGTGGAGATTG-3′; GAPDH anti-sense, 5′-TGCAGGATGCATTGCTGACA-3′. Internal control GAPDH was used to evaluate the relative expression levels of Bcl-2 and Bax. PCR products were analyzed on 1% agarose gels.

### 4.6. Immunoblot Analysis

Cells were washed twice with ice cold PBS, and total cell lysates were obtained by adding 50 or 100 mL of RIPA buffer (PBS, 1% NP-40, 0.5% sodium deoxycholate, and 0.1% SDS, containing fresh protease inhibitor cocktail) to the SH-SY5Y cells (1 × 10^6^ cells/mL) cultured in six-well plates. The whole cell lysate (15 µg/lane) was subjected to 10% SDS-PAGE. Proteins were transferred to PVDF membranes and fixed using an electroblotting apparatus (Biorad, Hercules, CA, USA). After blocking the membranes in TBS containing 0.1% Tween-20 and 5% dry milk at room temperature for 1 h, they were incubated with primary antibodies (1:1000) overnight followed by conjugated secondary antibodies of horseradish peroxidase (1:10,000) incubation (Santa Cruz, CA, USA) at room temperature for 1 h. The optical densities of antibody-specific bands were then analyzed with an Image Analyzer LAS-3000 (Fuji, Japan).

### 4.7. Caspase-3 Activity Assay

The Caspase-3 activity was detected using a Colorimetric Caspase-3 Assay Kit (Sigma-Aldrich, St. Louis, MO, USA) as per the protocol of manufacturer. The caspase-3 activity assay was determined as described previously [51]. The reaction mixture (total volume, 200 μL) was carried out in 96-well plate. After incubation of the mixtures at 37 °C for 90 min. The emission and excitation absorbance values were measured at wavelengths of 360 and 460 nm, respectively.

### 4.8. Flow Cytometric Detection of Apoptotic Cells

SH-SY5Y cells (1 × 10^6^ cells/well) were collected by centrifugation following MPP^+^ exposure for 60 h following by washing with ice-cold PBS twice. Cell pellets were resuspended in ice-cold 70% ethanol and fixed at 4 °C for 24–48 h, washed with PBS, and resuspended with DNA staining reagent around 1 mL containing 50 µg/mL RNase, 0.1% Triton X-100, 0.1 mM EDTA (pH 7.4), and 50 µg/mL PI. Staining was proceeded at 4 °C for 30 min [52]. Red fluorescence (DNA) was detected with a 563–607 nm band pass filter using a FACS Caliber flow cytometer (Becton Dickinson, San Jose, CA, USA). Ten thousand cells in each sample were analyzed. The percentage of apoptotic cells accumulating in the sub-G1 peak was calculated with Cell Quest software (Becton Dickinson, San Jose, CA, USA).

### 4.9. Mitochondrial Membrane Potential (ΔΨm) Measurement

Mitochondrial transmembrane potential was assayed using JC-1. The ability of retention of the JC-1 dye is correlated with the mitochondrial transmembrane potential. After MPP^+^ treatment, cells were incubated with DMEM containing 5 µL of JC-1 for 15 min at 37 °C, washed twice with 200 µL of assay buffer, and placed in 100 µL assay buffer. The fluorescent densities were calculated after the OD values were measured on a Microplate Reader (Tecan, Meilen, Zurich, Switzerland) at wavelength of 485 and 535 nm, respectively.

### 4.10. Statistical Analysis

All measurements were performed in triplicate and repeated three times using different samples. Results are presented as means ± SEM. The level of statistical significance was determined by analysis of variance (ANOVA) followed by Dunnett’s *t*-test for multiple comparisons. *p*-Values of less than 0.05 were considered as statistically significant.
